# Sagittal alignment in Japanese elderly people - how much can we tolerate as normal sagittal vertical axis?

**DOI:** 10.1186/1748-7161-10-S1-O34

**Published:** 2015-01-19

**Authors:** Tatsuya Yasuda, Tomohiko Hasegawa, Yu Yamato, Sho Kobayashi, Daisuke Togawa, Hideyuki Arima, Yukihiro Matsuyama

**Affiliations:** 1 Department of Orthopaedic Surgery Hamamatsu University School of Medicine, Japan

## Objectives

Spinal sagittal alignment has been reported that it is highly associated with health related QOL in adult spinal deformity patients. Optimal sagittal alignment is critical for the efficient function of the musculoskeletal system. In Japan as aging country, a number of elderly people with spinal deformity seems to be increasing. In general, sagittal alignment of elderly people is more forward than younger people. But there is no data to show the average sagittal alignment in elderly people. It is still unknown how much we can tolerate as normal sagittal vertical axis. The purpose of this study was to investigate the relationship between sagittal alignment and health related QOL in elderly volunteers.

## Materials and methods

707 volunteers with age over 60 were participated in this IRB approved study in Toei town of Aichi prefecture in Japan. Lateral radiographs of whole spine and pelvis in standing position were taken. Radiographic parameters were included sagittal vertical axis (SVA), pelvic incidence (PI) minus lumbar lordosis (LL) and pelvic tilt (PT). Oswestry Disability Index (ODI) was investigated as health related QOL assessment. These radiographic parameters were utilized to investigate the relationship with ODI.

## Results

Digitized radiographs were successfully evaluated in 653 volunteers (195 male, 495 female, average age 74). Average of SVA, PILL and PT were 51mm, 8.9 degrees and 19 degrees, respectively. There were significant correlations between each parameter (SVA/PI-LL: r=0.58, SAV/PT: r=0.45, PI-LL/PT: r=0.69). SVA had strongest correlation (r=0.36) with ODI score in the three sagittal modifier parameters in SRS-Schwab classification. Using linear regression analysis, SVA was 87mm when ODI score was 40%.

## Conclusion

Previous studies reported that SVA had significant correlation with health related quality of life. Schwab reported that threshold values for severe disability (ODI > 40) were SVA 47 mm or more. Therefore, sagittal malalignment was defined in SVA of over 50 mm in many reports. The average age of our study was older than their study (74 years vs 51 years). This study showed that the range of normal SVA is wider for elderly people.

